# Cross-Reaction or Co-Infection? Serological Discrimination of Antibodies Directed against Dugbe and Crimean-Congo Hemorrhagic Fever Orthonairovirus in Nigerian Cattle

**DOI:** 10.3390/v13071398

**Published:** 2021-07-19

**Authors:** Julia Hartlaub, Oluwafemi B. Daodu, Balal Sadeghi, Markus Keller, James Olopade, Daniel Oluwayelu, Martin H. Groschup

**Affiliations:** 1 Institute of Novel and Emerging Infectious Diseases, Friedrich-Loeffler-Institut, Suedufer 10, 17489 Greifswald–Insel Riems, Germany; julia.hartlaub@fli.de (J.H.); balal.sadeghi@fli.de (B.S.); markus.keller@fli.de (M.K.); 2 Department of Veterinary Microbiology, University of Ilorin, Ilorin 240103, Nigeria; daodu.ob@unilorin.edu.ng; 3 Department of Veterinary Anatomy, University of Ibadan, Ibadan 200284, Nigeria; jkayodeolopade@yahoo.com; 4 Department of Veterinary Microbiology, University of Ibadan, Ibadan 200281, Nigeria; ogloryus@yahoo.com

**Keywords:** DUGV, Dugbe orthonairovirus, CCHFV, Crimean-Congo hemorrhagic fever orthonairovirus, Nigeria, serology, cattle, specificity, sensitivity, cross-reactivity

## Abstract

Dugbe orthonairovirus (DUGV) and Crimean-Congo hemorrhagic fever orthonairovirus (CCHFV) are tick-borne arboviruses within the order *Bunyavirales*. Both viruses are endemic in several African countries and can induce mild (DUGV, BSL 3) or fatal (CCHFV, BSL 4) disease in humans. Ruminants play a major role in their natural transmission cycle. Therefore, they are considered as suitable indicator animals for serological monitoring studies to assess the risk for human infections. Although both viruses do not actually belong to the same serogroup, cross-reactivities have already been reported earlier—hence, the correct serological discrimination of DUGV and CCHFV antibodies is crucial. In this study, 300 Nigerian cattle sera (150 CCHFV seropositive and seronegative samples, respectively) were screened for DUGV antibodies via N protein-based ELISA, indirect immunofluorescence (iIFA) and neutralization assays. Whereas no correlation between the CCHFV antibody status and DUGV seroprevalence data could be demonstrated with a newly established DUGV ELISA, significant cross-reactivities were observed in an immunofluorescence assay. Moreover, DUGV seropositive samples did also cross-react in a species-adapted commercial CCHFV iIFA. Therefore, ELISAs seem to be able to reliably differentiate between DUGV and CCHFV antibodies and should preferentially be used for monitoring studies. Positive iIFA results should always be confirmed by ELISAs.

## 1. Introduction

Dugbe orthonairovirus (DUGV) is a zoonotic, tick-borne arbovirus (order *Bunyavirales*), which occurs widespread throughout Africa. In 1964, it was first isolated in Nigeria out of a pool of *Amblyomma variegatum* ticks [1]. Most of the isolates were obtained from ticks, but several strains have also been isolated from animals (cattle, wild rodents) and humans [1,2,3,4]. Ruminants seem to play a major role in the infection cycle, as most of the DUGV-positive ticks were collected from sheep, goats and cattle. Actually, DUGV is thought to be the most frequently isolated arbovirus in Nigeria [5]. However, all serological and virological investigations conducted on the virus occurred between 1964 and 1977 [1,6,7,8], and therefore, DUGV is quite a neglected virus in Nigeria nowadays. This might probably be due to its limited impact on animal and human health. No overt diseases have been observed in livestock, and reports of human infections, resulting in mild febrile illnesses, are scarce [1,3,4]. Hence, the influence on the public health and economic sector is rather low, and therefore, no increased awareness towards this arbovirus exists. 

However, DUGV might still be of significant importance, as a distant serological relationship to Crimean-Congo hemorrhagic fever orthonairovirus (CCHFV) is presumed. Although these viruses do not belong to the same serogroup, several studies have revealed serological cross-reactivities [9,10]. Sequence analyses have shown 43% and 16% amino acid differences between CCHFV and DUGV for the complete sequence of the S segment and partial genome sequences of the L segment, respectively [11,12]. In contrast to DUGV, the public health impact of CCHFV is not negotiable. Causing severe hemorrhagic fever with case fatality rates of 5–30% in humans, CCHFV is on the WHO R&D list of blueprint priority diseases [13]. To elucidate the current distribution of this BSL 4 agent and to assess the risk for human infections, serological monitoring studies involving ruminants are being conducted worldwide [14]. Due to their genetic and antigenic relationship, concerns arise that antibodies directed against DUGV might interfere with current CCHFV serological assays. If these antibodies led to false-positive CCHFV test results, the distribution and prevalence of CCHFV would be rather overestimated in regions where DUGV is also prevalent. In fact, Nigeria is a suitable representative for an African country, where both viruses co-exist. Recent studies have revealed CCHFV seroprevalence in Nigerian cattle (24% of 50 bovines) and CCHFV-IgG antibodies (10.6% of 1189 sera) in Nigerians, respectively [15,16]. In 2016, the first published CCHFV case diagnosed by RT-qPCR was reported [16].

Whereas in former studies, methods such as hemagglutination inhibition (HI) and complement fixation (CF) were utilized [9], only a few recent studies exist where ELISAs were employed to investigate putative cross-reactivities. Two studies were conducted in Africa to search for DUGV and CCHFV antibodies in cattle. Formalin or β-propiolactone inactivated virus stocks were coated on ELISA plates, and prevalence data for DUGV and CCHFV antibodies were compared. However, the obtained results were quite contradictory: whereas the authors from the study in the Central African Republic reported that 96% of the tested cattle sera with antibodies against CCHFV also reacted with the DUGV antigen, a study from South Africa revealed that only 7% of sera with CCHFV-reactive antibodies did also bind to the DUGV antigen [17,18]. Moreover, even if recombinant proteins were utilized, which are thought to be more specific than inactivated whole virus antigens, slight cross-reactivities were observed when the DUGV N protein was challenged with mono- or poly-specific CCHFV antisera in Western Blots and ELISAs [19].

Recently performed studies with immunized and experimentally infected sheep and cattle revealed that ruminants do not show any clinical signs nor develop viremia, detectable by RT-qPCR. However, a constant feature in both species was the generation of DUGV specific antibodies [20]. In the course of this study, we have developed several assays for the detection of these antibodies, namely an indirect ELISA based on recombinant N protein, an indirect immunofluorescence assay (iIFA) and a micro-virus neutralization test (mVNT). Furthermore, all serum samples were tested with currently used CCHFV diagnostic assays (FLI CCHFV In-house ELISA, Vector-Best ELISA, IDVet double antigen ELISA, Euroimmun iIFA) to investigate potential antibody cross-reactivities. Our data indicate that CCHFV ELISA systems were able to discriminate between these antibodies, whereas the immunofluorescence assay was not. However, these findings have to be reassured when sera following natural infections are analyzed, as these might actually yield higher levels of antibodies than artificially infected animals. Furthermore, only antibodies raised against one DUGV strain (IBAR1792) have been tested, but strain-specific antibodies against different currently circulating DUGV strains could also have an impact on cross-reactivities.

The aim of the presented work was to validate the new DUGV serological assays utilizing sera from Nigerian cattle, including the determination of ELISA cut-off values, as well as diagnostic specificities and sensitivities. We first pre-tested the sera for the presence of CCHFV antibodies with previously validated CCHFV serological assays. Out of this serum panel, we subsequently selected 150 CCHFV clearly seropositive and 150 definite seronegative samples, respectively, and analyzed them with our DUGV assays (ELISA, iIFA, mVNT). Our main interest in this project was the comparison of both groups and to evaluate whether the CCHFV antibody status has any influence on DUGV seroprevalence data and vice versa. 

In summary, serological assays for CCHFV (and DUGV) will serve solely as valuable tools for monitoring programs conducted in countries where both related viruses are endemic if antibodies to both viruses are reliably discriminated. The results obtained within this study will hopefully contribute to a more profound and justified interpretation of seroprevalence data in the fields of *Orthonairoviruses* in the future.

## 2. Materials and Methods

### 2.1. Sera: Collection and Selection

In 2018, cattle sera were collected from different local government areas in Kwara State, North-Central Nigeria. Samples were taken at abattoirs and farms (nomadic and semi-nomadic settlements). All sera were sent to Friedrich-Loeffler-Institut, Greifswald-Insel Riems, Germany for serological investigations. Before these analyses, samples were tested for the presence of CCHFV RNA (RT-qPCR), and only negative samples were further used for serological testing. Sera were first tested for the presence of CCHFV antibodies. For the presented study, out of these sera, 150 CCHFV seropositive (positive in IDVet ELISA, positive or doubtful in Vector-Best ELISA and FLI CCHFV In-house ELISA, respectively) and 150 seronegative (negative in all 3 CCHFV ELISAs) samples were randomly selected. 

### 2.2. DUGV Serological Assays

DUGV serological assays were performed as previously described [20].

1. **ELISA**: The indirect ELISA is based on recombinant DUGV N protein (bacterially expressed, DUGV strain ArD44313). After a blocking step, sera were added (1/20 dilution), and plates were incubated with an anti-bovine secondary antibody. The reaction was induced by TMB and stopped with 1M H_2_SO_4_. Corrected OD_450_ values were calculated (wells coated with antigen-wells without antigen), and the percentage of the samples in comparison to the positive control (immunized cattle) was determined. The nucleotide and amino acid sequence identities between the utilized DUGV strain for N protein expression and other sequenced DUGV strains are presented in Table 1.
2. **iIFA**: The indirect immunofluorescence assay is based on DUGV (IBAR1792) infected Vero E6 cells and non-infected control wells. After a blocking step, all sera (1/50 diluted) were incubated before the secondary antibody was added, and the fluorescence signal was evaluated. Sera were scored positive if a specific staining of DUGV infected cells was visible without non-specific compounds against non-infected Vero E6 cells.
3. **mVNT**: The micro-virus neutralization test was performed on SW13 cell monolayers in a 96-well format. Serial serum dilutions were incubated with 100 TCID_50_ DUGV (IBAR1792) and then transferred in duplicates to the prepared SW13 plates. Plates were stained with crystal violet after 7 days, and the cytopathic effect was evaluated. Neutralizing titers were calculated according to Behrens and Kaerber. Sera with titers ≥ 1/10 were scored positive.

### 2.3. CCHFV Serological Assays

1. IDVet double antigen ELISA (IDVet, Grables, France): This ELISA is based on recombinant CCHFV N protein (clade III) and was performed according to the manufacturers’ instructions. The percentage of the sample in comparison to the positive control was calculated (S/P%). Sera >30% were scored positive.
2. Vector-Best ELISA (Novosibirsk, Russia): This ELISA is based on CCHFV whole virus antigen (clade IV). A species-adapted protocol for bovines was performed as described before [21]. Optical density (OD) values were determined. Sera with values <0.3 and >0.5 were scored negative and positive, respectively. Doubtful results were in between.
3. FLI CCHFV In-house ELISA: This ELISA is based on recombinant CCHFV N Protein (clade V). A bovine-specific protocol was used [22]. The percentage of the sample in comparison to the positive control was calculated (S/P%). Sera <16% were scored negative and >19% positive. Doubtful results were in between.
4. Indirect immunofluorescence assay (Euroimmun, Luebeck, Germany): This assay is based on cells expressing GPC (glycoprotein precursor) and N proteins of CCHFV (clade III). A bovine-specific protocol was used [21]. Sera were scored positive if a specific fluorescence signal was detected in comparison to the signal detected for non-transfected cells.

### 2.4. Statistical Analysis

The area under the receiver operating characteristic (ROC) curve was used to determine the ELISA cut-off value. Sensitivity, specificity, positive predictive value (PPV) and negative predictive value (NPV) were evaluated. Statistical analyses were performed using MedCalc for Windows, version 19.4 (MedCalc Software, Ostend, Belgium). *p*-value < 0.05 was regarded as statistically significant.

The generalized linear model (GLM) was used for the determination of serological cross-reactivity. The percent agreement between the two assays was calculated using Pearson’s Chi-squared test. Statistical analysis was conducted with the SPSS software version 22.0 for Windows (IBM Corp., New York, NY, USA). *P*-value < 0.05 was considered statistically significant.

## 3. Results

No commercial DUGV serological diagnostic test, which could serve as a reference standard for the evaluation of the newly developed assays, is currently available. However, neutralization assays are, in general, rated as highly specific and therefore, the DUGV mVNT was set as a gold standard to validate the performance of the other assays (ELISA, iIFA).

### 3.1. Establishment and Validation of Indirect DUGV IgG ELISA 

**Figure 1:**
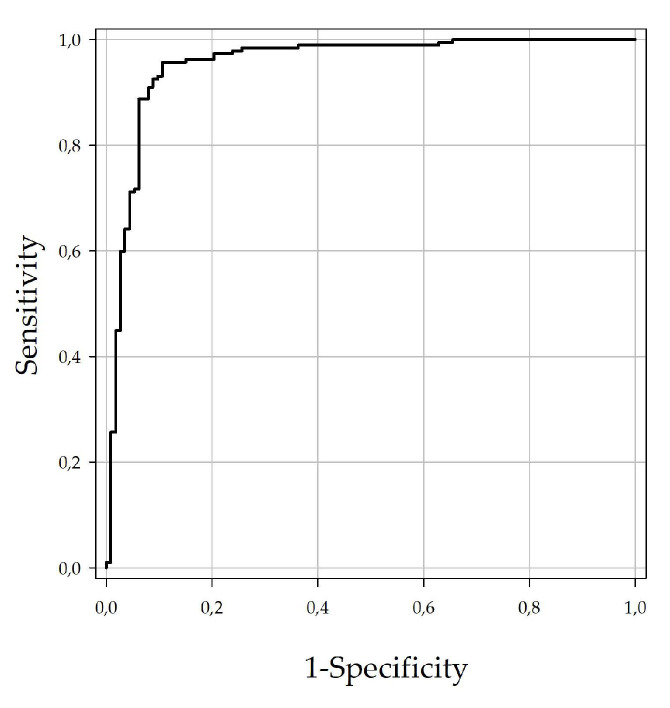
ROC analysis employing 300 Nigerian cattle sera: Diagnostic sensitivity of the DUGV ELISA is 95.7% (95% CI 91.7–98.1) and diagnostic specificity is 88.6% (95% CI 81.3–93.8) with AUC being 0.951 (*p*-value < 0.001).

**Figure 2:**
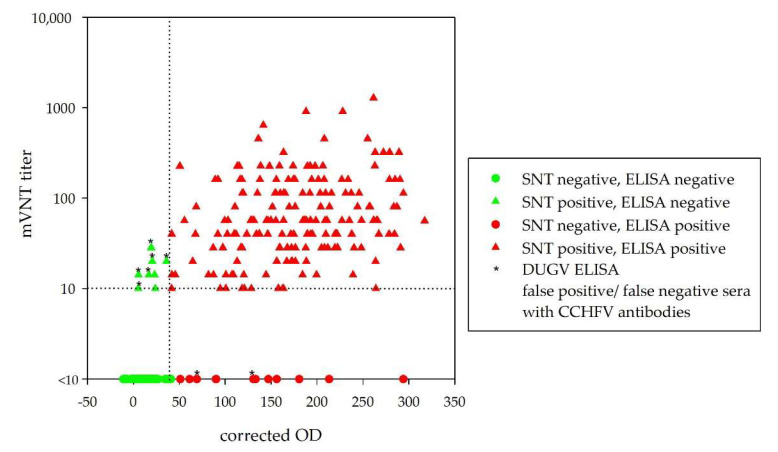
Correlation of mVNT titers and corrected OD values: only 8 sera were false-negative (green triangles) and 12 sera false-positive (red dots) out of 300 tested cattle sera. Six false-negative sera and two false-positive sera actually yielded CCHFV antibodies (marked with *).

All sera were tested with the DUGV ELISA, and receiver operating characteristic (ROC) analysis was performed for the determination of the ELISA cut-off value in regards to maximum sensitivity and specificity (Figure 1). The calculated cut-off (40.9%) led to a specificity of 88.6% and a sensitivity of 95.7%. Figure 2 illustrates the relationship between corrected OD values and mVNT titers for all 300 tested serum samples. 

**Figure 3:**
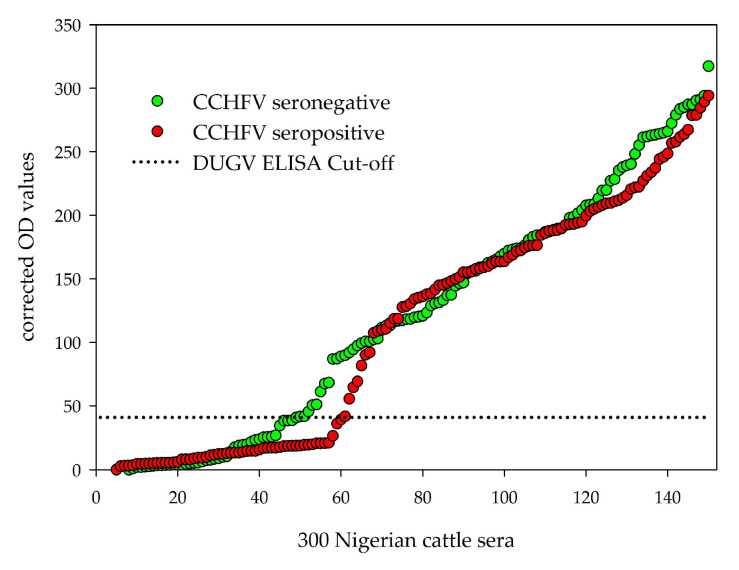
Corrected OD values for 300 Nigerian cattle sera: The distribution patterns between CCHFV seropositive sera (red dots) and CCHFV seronegative sera (green dots) do not apparently vary between each other.

**Figure 4:**
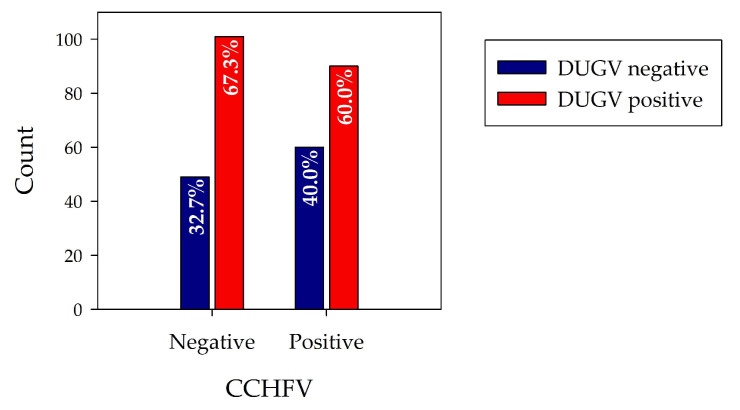
Cross-reactivity N protein-based ELISAs: The percentage of DUGV antibody-positive sera is slightly lower for the CCHFV seropositive samples, indicative of good serological discrimination of DUGV and CCHFV antibodies via ELISA.

In order to evaluate whether DUGV and CCHFV antibodies cross-react in the corresponding ELISAs, 150 clearly CCHFV seropositive and 150 clearly CCHFV seronegative serum samples were pre-selected and further analyzed with the DUGV mVNT and DUGV ELISA. Figure 3 visualizes the corrected OD values for both serum groups. Table 2 shows the cross-tabulation of DUGV and CCHFV data, and Figure 4 the corresponding bar chart.

When comparing DUGV seroprevalence data of CCHFV seropositive and CCHFV seronegative samples, it seems that the CCHFV status has no influence on the DUGV seroprevalence. Whereas 67% of CCHFV seronegative samples revealed anti-DUGV antibodies, the percentage was slightly lower for the CCHFV seropositive samples (60.0%). If antibodies directed against CCHFV led to positive results in the DUGV ELISAs, the fraction of DUGV-positive sera would be significantly higher for sera with positive CCHFV status, and no or extremely low numbers of complete DUGV seronegative sera would be expected. 

**Figure A1:**
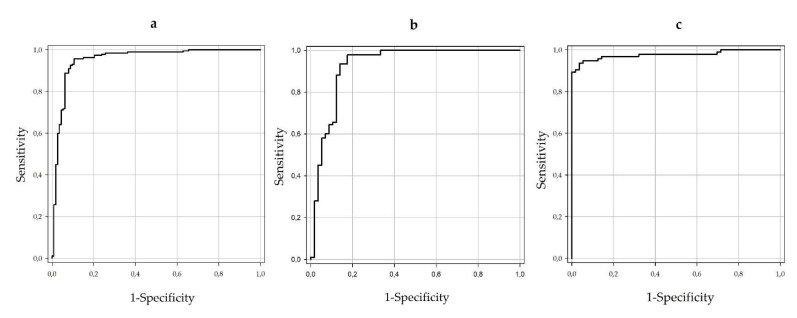
Comparative ROC analyses: Graph (**a**) shows the ROC curve for all 300 serum samples, graph (**b**) the 150 CCHFV seronegative samples and graph (**c**) the 150 CCHFV seropositive samples only.

ROC analysis employing the CCHFV seronegative samples only revealed reduced AUC (area under the curve) and specificity values compared to the calculation for all data or for CCHFV seropositive samples, respectively (Table 3, Appendix A Figure A1). The inclusion of sera with CCHFV antibodies does not decrease the specificity of the DUGV ELISA. Hence, these findings further support the assumption that the performance of our DUGV ELISA is not negatively influenced by the presence of CCHFV antibodies. 

Moreover, evaluation of cross-reactivity between CCHFV and DUGV was done with the generalized linear model. CCHFV seropositive and seronegative samples were used as independent variables, and samples ID and the interaction of CCHFV and DUGV were set as random effects. There was no association between the seropositive and seronegative CCHFV sera (*p* = 0.742) to detect antibodies against DUGV. Pearson’s Chi-squared test showed that there is no statistically significant correlation between the CCHFV antibody status and DUGV antibody presence (*p* = 0.218). These results confirmed that there is no statistically significant difference between CCHFV seropositive and seronegative samples in regards to DUGV antibody detection. 

### 3.2. Validation of DUGV and CCHFV Immunofluorescence Assays

#### 3.2.1. DUGV iIFA

**Figure 5:**
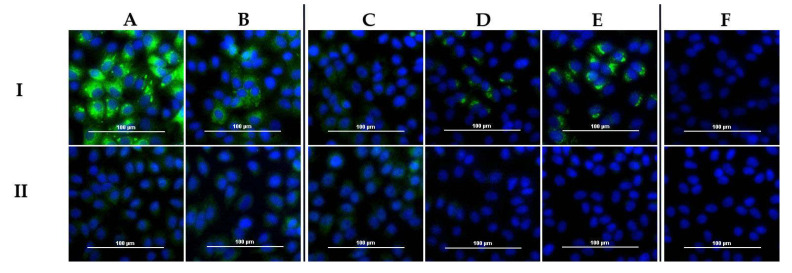
DUGV IFA: Cells infected with DUGV (**I**), as well as non-infected control wells (**II**), are depicted. Sera A and B represent DUGV seropositive samples, with serum A being higher seropositive than serum B. Sera C, D and E yielded CCHFV antibodies only. Whereas serum C is an example of a true-negative serum, sera D and E show specific staining of DUGV-infected cells. Serum F (CCHFV−/DUGV−) served as a negative control.

All cattle sera were analyzed with the DUGV iIFA (whole virus antigen). In Figure 5, results for the corresponding sera of Table 4 are depicted. Detailed serological data for these sera are available in Appendix A (Table A1). Not only DUGV antibody-positive sera led to a specific staining of DUGV-infected Vero E6 cells, but also DUGV antibody-negative sera, which yielded high amounts of CCHFV antibodies.

Diagnostic specificity, as well as the sensitivity of the DUGV iIFA, was calculated based on the DUGV mVNT data as a gold standard. The fraction of false-positive sera in the iIFA was 3-fold higher (18% vs. 6%) for CCHFV seropositive sera than for CCHFV seronegative samples (Table 5). Diagnostic sensitivity was identical for both groups, but diagnostic specificity was significantly lower (52% vs. 84%) for CCHFV seropositive sera (Table 6).

#### 3.2.2. CCHFV iIFA (Euroimmun)

A selection of Nigerian sera was tested with the species-adapted commercial CCHFV iIFA (Euroimmun, Luebeck, Germany), as it has previously been observed that ruminant sera following experimental DUGV immunizations, as well as infections, were cross-reactive in this assay [20].

**Figure 6:**
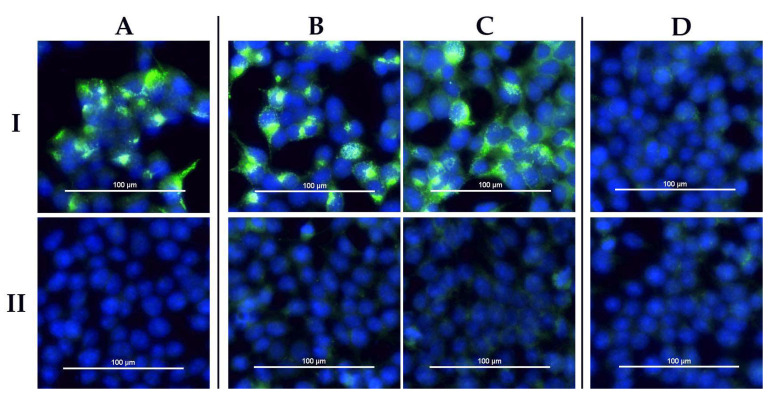
CCHFV IFA (Euroimmun, Lübeck): Cells expressing CCHFV GPC (**I**) and non-transfected control cells (**II**) are depicted. Serum A (CCHFV+/DUGV−) led to an undistinguishable fluorescent signal compared to sera B and C (CCHFV−/DUGV+). Serum D (CCHFV−/DUGV−) served as the negative control.

Figure 6 shows the results for the corresponding sera of Table 7 (detailed results are provided in Appendix A, Table A2). Cattle sera that tested positive for DUGV antibodies without the presence of CCHFV antibodies led to a highly specific staining of transfected cells. This signal could not be discriminated from true positive CCHFV antisera.

## 4. Discussion

DUGV and CCHFV, both members of the family *Nairoviridae*, are co-existing in Nigeria at present, as indicated by the serology data presented in this report. Serological assays for the detection of DUGV antibodies were established and validated utilizing 300 serum samples from Nigerian cattle. The major interest of this study was to investigate whether the CCHFV antibody status has any influence on DUGV antibody detection and vice versa.

DUGV was first isolated in Nigeria [1] and was thereafter thought to be the most often isolated arbovirus there. However, the last reports were published in the 1970s [6,7,8,23]. Around 50 years later, we were interested in whether DUGV was still present in Nigeria. Hence, we tested cattle sera for the presence of DUGV-specific antibodies as an indicator for DUGV circulation. Out of 300 sera, 187 samples revealed anti-DUGV antibodies by mVNT. Nearly two-thirds (62.3%) of the sampled cattle had a previous DUGV infection history, and we consequently assume that DUGV is still highly prevalent in Nigeria. Moreover, a substantial seroprevalence was also recorded for CCHFV (manuscript in preparation). In summary, both viruses are widely distributed in Nigeria, and therefore, this sample panel is suitable for studying the effects of co-infection and serological cross-reactions between DUGV and CCHFV. Moreover, both agents should be taken under consideration as differential diagnoses for human infections and diseases in Nigeria. DUGV (BSL 3) can induce a mild febrile illness, but one case was documented in South Africa with more severe clinical symptoms, including prolonged thrombocytopenia and signs of hemorrhagic fever. A monospecific rise of anti-DUGV antibodies without the detection of anti-CCHFV antibodies led to the assumption that this disease was indeed caused by DUGV [18]. The first confirmed human CCHFV infection in Nigeria was recorded in 2016 [16], implying that this BSL 4 agent may lead to further human cases in the future or might have caused undetected infections in the past. Therefore, the serological monitoring of animals (mainly ruminants) can contribute to a risk assessment concerning human CCHFV (and DUGV) infections. As cross-reactivities between both agents have been reported, our main focus was on the serological discrimination of these antibodies.

To validate the DUGV serological assays, the mVNT was set as a gold standard. All sera were then analyzed with the DUGV ELISA, and iIFA, and diagnostic specificity and sensitivity were calculated for each assay. Concerning the ELISA, ROC analysis revealed 95.7% sensitivity and 88.6% specificity for the determined cut-off value (40.9% of positive control). All false -negative sera displayed only weak mVNT titers between 1/10 and 1/20. The ELISA cut-off was significantly higher than the previously calculated cut-off employing 100 German cattle sera (mean + 3× standard deviation = 17.9%), underlining the need to include African reference sera when establishing such serological assays. When comparing corrected OD values for experimentally inoculated (DUGV strain: IBAR 1792) and naturally infected cattle, the mean value for all Nigerian sera (encountering sera with corrected OD values > 40.9%) was 174.1%, whereas four experimentally challenged animals only had corrected OD values of 28.7%, 33.1%, 79.4% and 101.6%, respectively [20]. It appears that there is no correlation between the CCHFV antibody presence and the DUGV ELISA results, as the cross-tabulation and comparative ROC analyses did not reveal an interrelation. In addition, the generalized linear model and Pearson Chi-squared calculations have not shown a statistically significant association. In summary, the new DUGV ELISA is a valuable tool for further serological investigations in demands of high-throughput testing and can be used instead of the time-consuming and labor-intensive mVNT, which moreover requires a BSL 3 facility.

All sera were also tested with the DUGV iIFA, and, in contrast to the results obtained with the ELISA, significant cross-reactivities were observed for CCHFV seropositive samples. The amount of DUGV seronegative sera, which tested false-positive in the iIFA, was 3-fold increased for CCHFV seropositive sera than for seronegative. We, therefore, do not recommend performing DUGV immunofluorescence assays. Moreover, a quarter of iIFA false-positive sera were actually CCHFV-negative, probably due to previous other (orthonairo) virus infections, which may also interfere in this assay. We have shown recently that antibodies to DUGV and Nairobi sheep disease orthonairovirus (NSDV) cross-react in the iIFA, whereas the corresponding ELISAs detected the antibodies in a species-specific way [24]. The investigation of cross-reactivities to Kupe orthonairovirus (KUPV) should be part of future research.

The other way round, DUGV seropositive samples also led to positive results in the species-adapted commercial CCHFV immunofluorescence assay. This was already demonstrated for experimentally infected calves [20]. As only the GPC (glycoprotein precursor) expressing cells led to positive results, cross-reactivities between DUGV and CCHFV are most likely based on similar epitopes for the envelope glycoproteins. Therefore, these findings do not contradict our observations that N protein-based ELISAs can reliably discriminate between DUGV and CCHFV antibodies. Serological cross-reactions in immunofluorescence assays for orthonairoviruses have already been reported earlier [9]. In this study, only bovine sera were analyzed. However, it is likely that similar cross-reactivities in immunofluorescence assays also apply for other animal sera (e.g., ovine, caprine) or even human sera. This pertains not only to Nigeria but also to further African countries, where both viruses co-exist.

Concerning cross-neutralizing epitopes, the neutralizing potential of CCHFV antibodies for DUGV was included in this study. Six sera (out of 60 CCHFV seropositive sera below the DUGV ELISA cut-off) displayed weak mVNT titers. These findings do not support the presence of cross-neutralizing antibodies, as CCHFV seronegative samples (2 out of 49) also revealed DUGV neutralizing antibodies with negative results in the DUGV ELISA. Therefore, we assume that the CCHFV immune status does not influence the susceptibility to a consequent DUGV infection; this will probably also be true vice versa. However, it cannot be fully excluded that there might be an influence on the pathogenesis during a following heterologous infection, e.g., a shorter viremic state due to a faster immune response, particularly if cross-reactivities between the glycoproteins are presumed (see CCHFV iIFA results), which are actually thought to be the target for neutralizing antibodies [25,26]. Nevertheless, this hypothesis should be neglected as even homologous reinfections with CCHFV have been documented [27,28,29]. 

Furthermore, the neutralizing antibodies in DUGV-ELISA negative sera might be the result of earlier infections with other orthonairoviruses. Recently, we have shown that NSDV antisera can also lead to weak positive results in the DUGV mVNT. However, NSDV has not been isolated in Nigeria yet, and therefore, we assume that the DUGV mVNT is still a reliable gold standard for the detection of DUGV antibodies [24]. The impact of cross-neutralizing antibodies on KUPV has not been investigated so far. In parallel, increased sensitivity of the DUGV mVNT in comparison to the novel DUGV ELISA might be another suitable explanation for the eight false-negative serum samples. Indeed, recent studies have revealed that neutralization assays were slightly more sensitive than ELISAs for the detection of DUGV and NSDV antibodies [24].

In conclusion, the ELISA and mVNT results clearly emphasize the co-circulation of DUGV and CCHFV, rather than cross-reactions between the two viruses, in Nigerian cattle. 

## Figures and Tables

**Table 1:** N protein homologies of known DUGV strains.

		Nucleotides		
		ArD 44313	IbH 11480	IbAr 1792
**Amino acids**	ArD 44313	X	99.04%	99.24%
	IbH 11480	99.59%	X	99.52%
	IbAr 1792	99.79%	99.79%	X

(analysis: available GenBank sequences, calculation performed by Geneious).

**Table 2:** Cross-tabulation table ELISA (CCHFV × DUGV).

			DUGV		Total
			Neg	Pos	
CCHFV	Neg	Count	49	101	150
		% within CCHFV	32.7%	67.3%	100.0%
	Pos	Count	60	90	150
		% within CCHFV	40.0%	60.0%	100.0%

**Table 3:** Comparative ROC analysis.

	All Data	CCHFV Seronegative	CCHFV Seropositive
AUC	0.95	0.93	0.98
Specificity	88.6%	82.5%	94.6%
Sensitivity	95.8%	97.8%	94.7%

**Table 4:** Corresponding data to Figure 5.

Diagnostic Assay		A (1077)	B (1151)	C (568)	D (359)	E (642)	F (980)
DUGV	DUGV ELISA	+ +	+	−	−	−	−
	DUGV mVNT	+ +	+	−	−	−	−
CCHFV	IDVet ELISA	−	−	+	+ +	+ +	−
	Vector−Best ELISA	−	−	+	+ +	+ +	−
	In−house ELISA	−	−	+ +	+	+ +	−

**Table 5:** Comparison of DUGV iIFA and DUGV mVNT.

**CCHFV seronegative samples**				
		**mVNT**		
		+	−	Total
iIFA	+	58%	6%	64%
	−	4%	32%	36%
	Total	62%	38%	100%
**CCHFV seropositive samples**				
		**mVNT**		
		**+**	**−**	**Total**
iIFA	+	59%	18%	77%
	−	3%	19%	23%
	Total	63%	37%	100%

**Table 6:** Specificity and sensitivity of DUGV iIFA.

	CCHFV		
	+	−	Total
Sensitivity	94%	95%	94%
Specificity	84%	52%	68%

**Table 7:** Corresponding serological data to Figure 6.

Diagnostic Assay		A (390)	B (571)	C (526)	D (497)
DUGV	DUGV ELISA	−	+ +	+ +	−
	DUGV mVNT	−	+	+	−
CCHFV	IDVet ELISA	+ +	−	−	−
	Vector−Best ELISA	+ +	−	−	−
	In−house ELISA	+	−	−	−

## Data Availability

The data presented in this study are available within this manuscript, Hartlaub et al., Viruses.

## Appendix A

**Table A1:** Detailed version of Table 4 in the main section.

Diagnostic Assay		unit	A (1077)	B (1151)	C (568)	D (359)	E (642)	F (980)
DUGV	DUGV ELISA	S/P%	293%	103%	13%	0%	12%	19%
	DUGV mVNT	ND_50_	321	57	<10	<10	<10	<10
CCHFV	IDVet ELISA	S/P%	7%	10%	119%	201%	235%	8%
	Vector-Best ELISA	OD	0.29	0.21	1.27	1.81	2.55	0.4
	In-house ELISA	S/P%	16%	8%	155%	97%	133%	−8%

S/P%: sample to positive ratio, ND_50_: 50% neutralization dose, OD: optical density.

**Table A2:** Detailed version of Table 7 in the main section.

Diagnostic Assay		Unit	A (390)	B (571)	C (526)	D (497)
DUGV	DUGV ELISA	S/P%	−9%	265%	262%	−9%
	DUGV mVNT	ND_50_	<10	57	57	<10
CCHFV	IDVet ELISA	S/P%	196%	5%	9%	4%
	Vector-Best ELISA	OD	1.64	0.31	0.22	0.31
	In-house ELISA	S/P%	22%	10%	7%	4%

S/P%: sample to positive ratio, ND_50_: 50% neutralization dose, OD: optical density.
